# Vaccine-induced immune responses against both Gag and Env improve control of simian immunodeficiency virus replication in rectally challenged rhesus macaques

**DOI:** 10.1371/journal.ppat.1006529

**Published:** 2017-07-21

**Authors:** Mauricio A. Martins, Young C. Shin, Lucas Gonzalez-Nieto, Aline Domingues, Martin J. Gutman, Helen S. Maxwell, Iris Castro, Diogo M. Magnani, Michael Ricciardi, Nuria Pedreño-Lopez, Varian Bailey, Dillon Betancourt, John D. Altman, Matthias Pauthner, Dennis R. Burton, Benjamin von Bredow, David T. Evans, Maoli Yuan, Christopher L. Parks, Keisuke Ejima, David B. Allison, Eva Rakasz, Glen N. Barber, Saverio Capuano, Jeffrey D. Lifson, Ronald C. Desrosiers, David I. Watkins

**Affiliations:** 1 Department of Pathology, University of Miami, Miami, Florida, United States of America; 2 Department of Microbiology and Immunology, University of Miami, Miami, Florida, United States of America; 3 Department of Microbiology and Immunology, Emory University, Atlanta, Georgia, United States of America; 4 Department of Immunology and Microbiology, IAVI Neutralizing Antibody Center, Center for HIV/AIDS Vaccine Immunology and Immunogen Discovery (CHAVI-ID), The Scripps Research Institute, La Jolla, California, United States of America; 5 Department of Pathology and Laboratory Medicine, University of Wisconsin–Madison, Madison, Wisconsin, United States of America; 6 Wisconsin National Primate Research Center, University of Wisconsin–Madison, Madison, Wisconsin, United States of America; 7 International AIDS Vaccine Initiative, AIDS Vaccine Design and Development Laboratory, Brooklyn, New York, United States of America; 8 Section on Statistical Genetics, Department of Biostatistics, University of Alabama at Birmingham, Birmingham, Alabama, United States of America; 9 Department of Cell Biology, University of Miami, Miami, Florida, United States of America; 10 AIDS and Cancer Virus Program, Leidos Biomedical Research, Inc., Frederick National Laboratory for Cancer Research, Frederick, Maryland, United States of America; Emory University, UNITED STATES

## Abstract

The ability to control lentivirus replication may be determined, in part, by the extent to which individual viral proteins are targeted by the immune system. Consequently, defining the antigens that elicit the most protective immune responses may facilitate the design of effective HIV-1 vaccines. Here we vaccinated four groups of rhesus macaques with a heterologous vector prime/boost/boost/boost (PBBB) regimen expressing the following simian immunodeficiency virus (SIV) genes: *env*, *gag*, *vif*, *rev*, *tat*, and *nef* (Group 1); *env*, *vif*, *rev*, *tat*, and *nef* (Group 2); *gag*, *vif*, *rev*, *tat*, and *nef* (Group 3); or *vif*, *rev*, *tat*, and *nef* (Group 4). Following repeated intrarectal challenges with a marginal dose of the neutralization-resistant SIVmac239 clone, vaccinees in Groups 1–3 became infected at similar rates compared to control animals. Unexpectedly, vaccinees in Group 4 became infected at a slower pace than the other animals, although this difference was not statistically significant. Group 1 exhibited the best post-acquisition virologic control of SIV infection, with significant reductions in both peak and chronic phase viremia. Indeed, 5/8 Group 1 vaccinees had viral loads of less than 2,000 vRNA copies/mL of plasma in the chronic phase. Vaccine regimens that did not contain *gag* (Group 2), *env* (Group 3), or both of these inserts (Group 4) were largely ineffective at decreasing viremia. Thus, vaccine-induced immune responses against both Gag and Env appeared to maximize control of immunodeficiency virus replication. Collectively, these findings are relevant for HIV-1 vaccine design as they provide additional insights into which of the lentiviral proteins might serve as the best vaccine immunogens.

## Author summary

There is still some uncertainty as to which HIV-1 proteins should be targeted by vaccine-induced immune responses. Indeed, studies of primary HIV-1 and SIV infections have reported that T-cell responses against different viral proteins can influence viral replication levels. To understand which antigens elicit the antiviral responses best able to control viral replication, we vaccinated rhesus macaques with different combinations of SIV antigens and then challenged them intrarectally with a pathogenic SIV clone using a regimen intended to mimic physiologically relevant human exposures to HIV-1. Vaccination with Env, Gag, Vif, Rev, Tat, and Nef did not prevent infection but resulted in substantial control of viremia in 5/8 infected vaccinees. Importantly, vaccine-induced immune responses against Env and Gag were required for this outcome. Curiously, macaques vaccinated with Rev, Tat, Nef, and Vif acquired infection at a slower rate than did the control group, although this difference was not statistically significant. Together, these results suggest that expanding the number of vaccine-encoded antigens beyond Env and Gag might improve control of viral replication.

## Introduction

The development of a prophylactic vaccine against HIV-1 has proven exceedingly difficult. While most successful vaccines rely on the induction of neutralizing antibodies (nAbs) to protect against infection, eliciting such responses against HIV-1 has been hampered by several aspects of the lentivirus Env glycoprotein [1]. The *env* gene of both HIV and simian immunodeficiency virus (SIV) encodes a gp160 precursor protein that is post-translationally cleaved into two subunits, gp120 and gp41. Dimers of these cleavage products assemble into trimers to ultimately form the native Env spike. HIV-1’s resistance to neutralization stems from several factors, including the inaccessibility of neutralizing epitopes in the native trimer, its poorly immunogenic glycan shield, and the enormous *env* sequence diversity of circulating isolates [1]. Despite these barriers, a fraction of infected individuals develop antibodies capable of potently neutralizing a wide spectrum of HIV-1 isolates [1], indicating that it is possible to harness the human immune system to mount such responses.

The RV144 “Thai trial” remains the only report of vaccine-mediated reduction (albeit modest) in HIV-1 infection rates [2], but this result remains controversial [3, 4]. A subsequent investigation of immune correlates of protection revealed that vaccine-induced IgG binding antibodies against the Env variable regions 1 and 2 (V1/V2) were associated with reduced HIV-1 acquisition [5], implying that antibody functions other than neutralization might have been responsible for the apparent protection reported in RV144. Recent monkey studies have also linked vaccine-elicited binding antibodies directed against V1/V2 to protection against mucosal infection with the biological isolate SIVmac251 [6–9]. However, except for live-attenuated SIV vaccines [10], no vaccine regimen has been able to prevent mucosal infection with the SIVmac239 clone, perhaps due to the unusual resistance of its Env protein to neutralization [11–15].

Given the difficulty in engendering broadly reactive anti-HIV-1 nAbs by vaccination, considerable efforts have been devoted to the development and optimization of vaccine regimens aimed at eliciting cellular immunity against HIV-1 since T-cell responses have been associated with control of viral replication [16]. Two factors must be considered when designing vaccines for the induction of cellular immunity: the vector platform and which inserts to use. In terms of the former, most immunization protocols have relied on DNA plasmids or replication-defective viral vectors to deliver HIV-1 or SIV genes for eliciting T-cell responses [17–20]. Since these approaches provide only transient Ag production, they favor the induction of central memory T-cell (T_CM_) responses [21, 22]. Although vaccine-induced T_CM_ have been shown to confer some measure of control of SIV replication [22], they rely on anamnestic expansion to produce enough effector cells to suppress viral replication [21]. Previous mouse studies have shown that this process can take several days after infection [23]. Alarmingly, however, SIV has been shown to cross the rectal epithelium and reach lymphoid tissues of rectally-challenged rhesus monkeys as early as 4 hr after virus exposure [24]. Thus, the kinetics of a vaccine-induced T_CM_-based response might be too slow to cope with the dynamism and fast pace of lentivirus infection.

In contrast to T_CM_, effector memory T-cells (T_EM_) are poised for immediate effector function and do not need antigen (Ag) restimulation to exert cytotoxic activity [21]. Additionally, T_EM_ recirculate through mucosal tissues where the majority of immunodeficiency virus transmissions take place [21]. These antiviral properties have prompted the development of immunization protocols that provide recurrent Ag exposure since this type of immune stimulation drives CD8+ T-cell differentiation toward the T_EM_ phenotype.

One strategy that has shown promise in pre-clinical SIV trials employs a fibroblast-adapted strain (68–1) of the persistent β-herpesvirus rhesus cytomegalovirus (RhCMV) to deliver SIV Ag [25, 26]. Although RhCMV/SIV vaccination does not protect monkeys from infection with SIVmac239, 50% of vaccinees manifest early control of viral replication and eventually clear the infection [27]. Of note, the efficacy of this vaccine regimen likely depends on the ability of 68-1-based RhCMV/SIV vectors to engender CD8+ T_EM_ restricted by major histocompatibility complex class (MHC)-II and/or non-classical MHC-I molecules [28, 29].

Experiments conducted in mice have shown that the iterative waves of Ag delivered as part of heterologous prime/boost/boost (PBB) vaccine regimens can also generate CD8+ T_EM_ responses [30, 31]. To explore this approach in nonhuman primates, we designed a new mixed modality immunization protocol comprising an electroporated recombinant DNA (EP rDNA) prime followed by sequential vaccinations with recombinant (r) adenovirus type-5 (rAd5) and vesicular stomatitis virus (rVSV) vectors to elicit SIV-specific immune responses. Since it is unclear if sequential boosting with replication-impaired vectors can maintain high frequency CD8+ T_EM_ for long periods of time, we incorporated a fourth and final boost into this immunization protocol using rhesus monkey rhadinovirus (RRV)-based vectors. RRV is a γ2-herpesvirus and, similar to RhCMV, establishes a persistent infection in rhesus macaques [32]. However, in contrast to RhCMV, rRRV/SIV vaccination has been shown to induce classical MHC-I-restricted CD8+ T_EM_ responses [33].

Having decided on an EP rDNA/rAd5/rVSV/rRRV PBBB regimen to elicit SIV-specific immune responses, we set out to determine which of the various lentivirus gene products might be the most efficacious in this immunization protocol. The choice of which viral Ag should serve as the targets of vaccine-induced cellular immunity has been a contentious issue, largely because T-cell responses against different viral proteins have been linked to discordant virologic outcomes in chronically HIV-1-infected individuals [34, 35]. For instance, broad CD8+ T-cell responses against Env, or accessory and regulatory proteins as a whole, have been associated with higher viral loads (VLs), whereas recognition of multiple epitopes in Gag has correlated with lower viremia [34]. Although these correlations were identified in cross sectional studies and therefore do not necessarily imply causation, they illustrate how the selection of T-cell immunogens for HIV-1 vaccines is not straightforward. Indeed, it is hard to argue against eliciting Env-specific immune responses in light of recent SIV vaccine trials showing that Env is required and sufficient for preventing SIV infection in rectally-challenged monkeys [6, 36]. Furthermore, while Gag has features of a useful immunogen [16, 37, 38], a Gag-only vaccine is unlikely to afford substantial control of viral replication in the event of HIV-1 infection. Lastly, T-cell responses against accessory and regulatory proteins may not correlate with reduced VLs in HIV-1-infected patients but they can result in significant control of SIV infection in vaccinated rhesus macaques [39, 40]. Curiously, T-cell responses against the accessory protein Vif have also been linked to lower infection risk in an HIV-1 preexposure prophylaxis trial [41]. Given these uncertainties, additional investigation is needed to define the lentivirus Ag that elicit the most protective immune responses.

Here we explored how the selection of SIV immunogens impacts vaccine efficacy. We vaccinated four groups of Indian rhesus macaques with an EP rDNA/rAd5/rVSV/rRRV PBBB regimen encoding different sets of SIV Ag and subsequently challenged them, alongside a group of sham-immunized control animals, intrarectally with SIVmac239. In comparing vaccine immunogenicity and efficacy among the four groups, we made several observations that might be relevant for the design of HIV-1 vaccine strategies.

## Results

**Fig 1:**
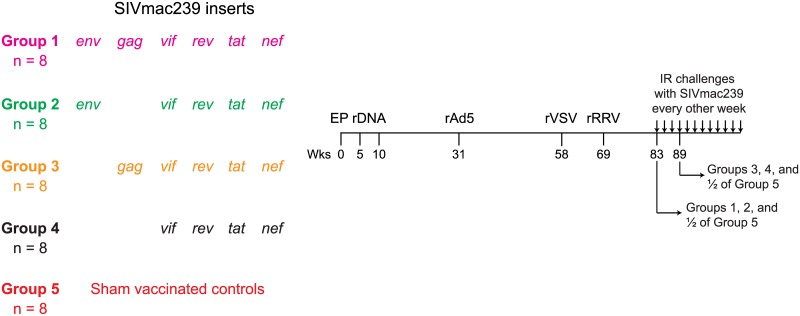
Experimental design. Eight Indian rhesus macaques in each of Groups 1–5 were vaccinated with an electroporated (EP) rDNA prime followed by sequential boosts with rAd5, rVSV, and rRRV vectors. This regimen delivered four different sets of SIVmac239 antigens. Group 1 was vaccinated with *env*, *gag*, *tat*, *rev*, *nef* and *vif*; Group 2 was vaccinated with *env*, *tat*, *rev*, *nef* and *vif*; Group 3 was vaccinated with *gag*, *tat*, *rev*, *nef* and *vif* and Group 4 was vaccinated with only *tat*, *rev*, *nef* and *vif*. Group 5 was sham-vaccinated with the same vectors used in Groups 1–4, except that they either lacked any inserts or expressed irrelevant genes. Macaques were primed with rDNA at 0, 5 and 10 wks post initiation of the regimen. rAd5 was delivered at week 31 and rVSV at week 58. The final rRRV boost occurred at wk 69. For logistical reasons, macaques in Groups 1, 2, and half of the monkeys in Group 5 were challenged for the first time at wk 83 post rRRV. Macaques in Groups 3, 4, and the remaining control animals in Group 5 were challenged for the first time at wk 89 post rRRV. The challenge regimen consisted of IR exposures to 200 TCID_50_ of SIVmac239 every other week.

A total of 32 Indian rhesus macaques were vaccinated with an EP rDNA/rAd5/rVSV/rRRV vaccine regimen encoding SIVmac239 genes. These animals were subdivided into four groups depending on the SIV antigens delivered by the PBBB regimen (Fig 1). The vaccinees in Group 1 (n = 8) were immunized with *env*, *gag*, *vif*, *rev*, *tat*, and *nef*; those in Group 2 (n = 8) received *env*, *vif*, *rev*, *tat*, and *nef*; macaques in Group 3 (n = 8) were vaccinated with *gag*, *vif*, *rev*, *tat*, and *nef*, while those in Group 4 (n = 8) were immunized with a more restricted set of immunogens, comprising *vif*, *rev*, *tat*, and *nef*. The Group 5 macaques (n = 8) were sham vaccinated with empty constructs or vectors encoding irrelevant inserts and served as the controls for this experiment. To facilitate monitoring of SIV-specific CD8+ T-cells by fluorochrome-labeled MHC-I tetramer staining, each group contained three or four monkeys that were positive for the MHC-I alleles Mamu-A*01 or Mamu-A*02 (Table 1). None of the animals in this experiment expressed the elite control-associated alleles Mamu-B*08 or Mamu-B*17.

**Table 1:** Animal characteristics.

Exptl group	Animal ID	MHC class I	Age (yrs)	Gender	TRIM5 genotype	RRV serology
Group 1	r09046	Mamu-A*01	4.4	Female	*TFP/CypA*	–
	r01007	Mamu-A*02	12.7	Male		+
	r03085	Mamu-A*02	10.3	Female		–
	r04017	Mamu-A*02	9.7	Female		+
	r06002		7	Female		–
	r06007		7	Female		–
	r08058		5	Male		–
	r10062		3.8	Female		–
Group 2	r09047	Mamu-A*01	4.4	Female		–
	r04106	Mamu-A*01	9.1	Female		–
	r04134	Mamu-A*02	9	Female		–
	r04074	Mamu-A*02	9.3	Female		+
	r08056		5	Male		IND
	r06030		6.9	Female		+
	r09017		4.6	Female		–
	rhAX27		14.3	Male		+
Group 3	r08061	Mamu-A*01	4.9	Male		–
	r07003	Mamu-A*02	6.7	Female		–
	r07007	Mamu-A*02	6.2	Female		+
	r06023	Mamu-A*02	6.9	Female		+
	rhBE56		5.2	Female		–
	r09004		4.8	Male		–
	r08027		5.3	Male		+
	r08030		5.3	Male	*TFP/Q*	–
Group 4	r09009	Mamu-A*01	4.7	Male		IND
	r08031	Mamu-A*02	5.3	Male	*TFP/Q*	+
	rh1994	Mamu-A*02	15.5	Female		+
	r04081	Mamu-A*02	9.2	Female		+
	r09002		4.8	Male		IND
	r06029		6.9	Female	*TFP/TFP*	IND
	rhBF18		4.3	Female		–
	rhBF24		4.3	Female	*TFP/TFP*	–
Group 5	r04156	Mamu-A*02	8.9	Female		+
	r02116		11.1	Female		+
	r05027		8.4	Female		+
	r09027		4.5	Female		+
	rh2313	Mamu-A*01	9.6	Male	*TFP/TFP*	+
	r05092	Mamu-A*01	8	Female		+
	r08037		5.2	Male		+
	r08040		5.2	Female		+

IND, indeterminate.

**Fig 2:**
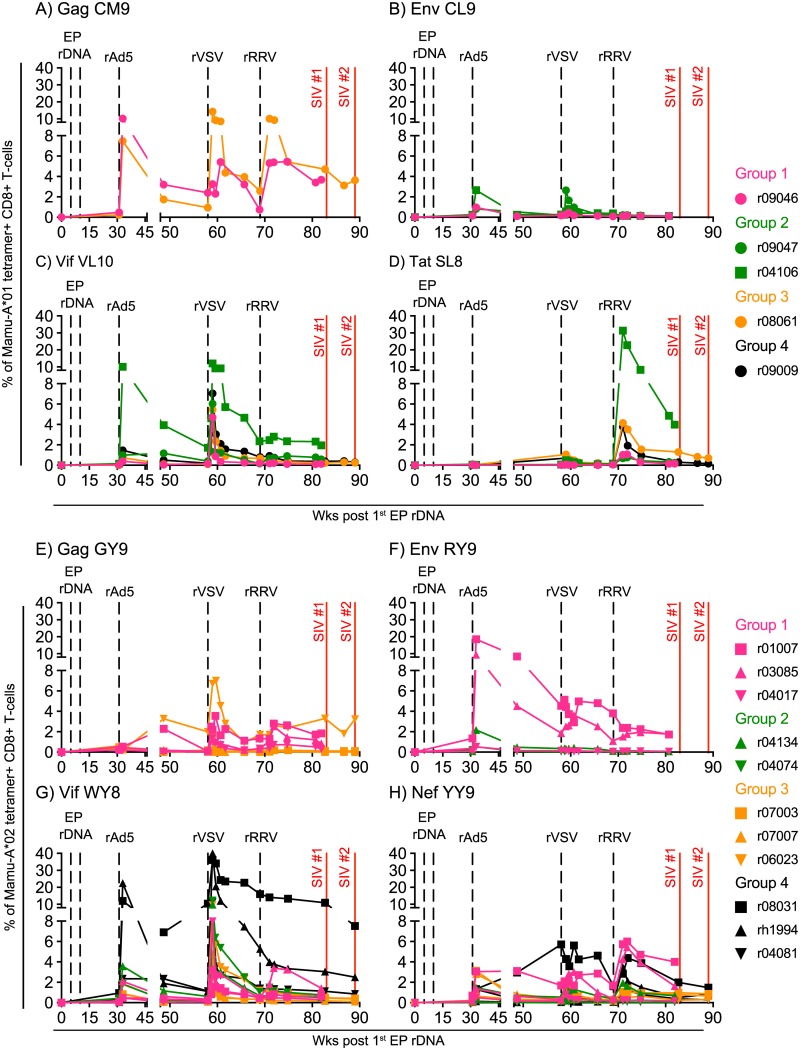
Vaccine-induced CD8+ T-cell responses directed against Mamu-A*01- or Mamu-A*02-restricted epitopes in macaques in Groups 1–4. Four macaques in each of Groups 1–4 expressed either Mamu-A*01 or Mamu-A*02 (Table 1). Using the appropriate fluorochrome-labeled MHC-I tetramers for each group, we monitored the ontogeny of vaccine-induced CD8+ T-cell responses specific for SIV epitopes restricted by Mamu-A*01 (A-D) and Mamu-A*02 (E-H). Each panel shows the magnitude of vaccine-induced CD8+ T-cells detected by individual MHC-I tetramers in animals in Groups 1–4. The following Mamu-A*01-restricted epitopes were evaluated: A) Gag CM9 (aa 181–189), B) Env CL9 (aa 233–241), C) Vif VL10 (aa 100–109), and D) Tat SL8 (aa 28–35). The following Mamu-A*02-restricted epitopes were evaluated: E) Gag GY9 (aa 71–79), F) Env RY9 (aa 296–304), G) Vif WY8 (aa 97–104), and H) Nef YY9 (aa 159–167). The times of each vaccination (vertical dashed black lines) and when the two SIVmac239 IR challenge rounds were started (vertical solid red lines) are shown in each graph. Macaques in Groups 1, 2, 3, and 4 are color coded in pink, green, beige, and black, respectively.

Both the rDNA and rAd5 vectors were delivered intramuscularly. However, in an attempt to elicit systemic immunity and direct vaccine-elicited CD8+ T-cells to relevant sites of lentivirus transmission and amplification, we delivered the rVSV vectors via both the intravenous (IV) and intrarectal (IR) routes. Curiously, while the rVSV boost increased the frequency of Vif-specific CD8+ T-cells in nearly all animals–especially those in Group 4 (Fig 2C and 2G), it had little effect on the levels of vaccine-elicited CD8+ T-cells targeting Env, Nef, and Tat (Fig 2B, 2D, 2F and 2H). The rVSV boost also augmented the size of the Gag CM9-specific CD8+ T-cell response in the Mamu-A*01+ Group 3 vaccinee r08061, but it had little effect on the response detected in the Group 1 animal r09046 (Fig 2A). This preferential expansion of CD8+ T-cells directed against Vif epitopes may have been due to the simultaneous administration of two rVSV/*vif* vectors to the macaques in Groups 1–4 (see Materials and methods).

The rRRV vectors were also co-delivered via the IV and IR routes and had a modest effect on the magnitude of SIV-specific CD8+ T-cell responses following vaccination. The Mamu-A*01+ Group 2 vaccinee r04106 was an exception since >30% of its peripheral CD8+ T-cells targeted the Tat SL8 epitope at week (wk) 2 post the rRRV boost (Fig 2D). One possible reason for the relatively poor expansion of SIV-specific CD8+ T-cells observed after the rRRV boost is that T-cell immunity engendered by the previous vaccinations may have limited the take of the rRRV vectors. Since these rRRV constructs are live herpesviruses, they need to infect and replicate in host cells in order to produce SIV antigens. Given that we delivered rRRV as the final viral vector boost, CD8+ T-cell responses against vaccine inserts generated by the EP rDNA, rAd5, and rVSV immunizations could have eliminated rRRV-infected cells in some of the animals before the establishment of a productive infection. Additionally, since several animals in Groups 1–4 were already naturally infected with RRV at the time of the rRRV vaccination (Table 1), pre-existing immunity to RRV antigens could also have decreased the take of the rRRV vectors in those monkeys.

**Fig 3:**
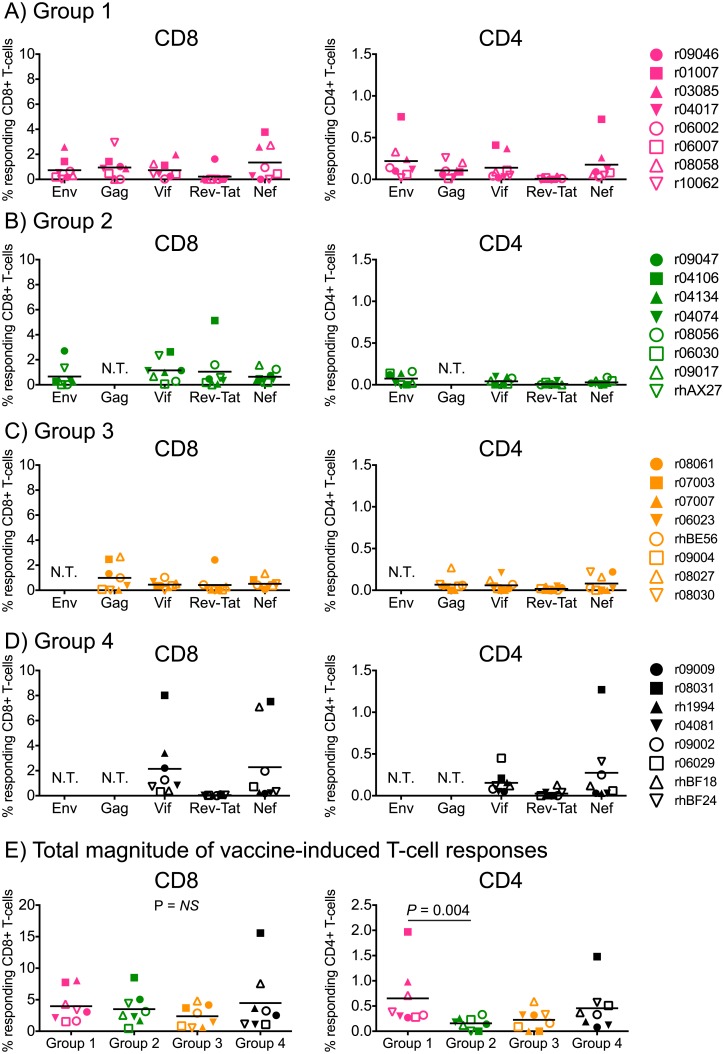
ICS analysis of vaccine-induced T-cell responses in Groups 1–4 at the time of SIV challenge. CD8+ and CD4+ T-cell responses were measured in PBMC by ICS using pools of peptides (15mers overlapping by 11 aa) spanning the appropriate SIVmac239 proteins. Peptides spanning the Rev and Tat proteins were tested together in the same in tubes. The percentages of responding CD4+ or CD8+ T-cells displayed in all panels were calculated by adding the frequencies of positive responses producing any combination of three immunological functions (IFN-γ, TNF-α, and CD107a). The magnitude and specificity of vaccine-induced CD8+ and CD4+ T-cell responses are shown for Group 1 (A), Group 2 (B), Group 3 (C), and Group 4 (D). The Kruskal-Wallis test was used for these comparisons and no difference was detected for CD8+ T-cell responses (*P* = 0.63). A significant difference in CD4+ T-cell responses was detected by this approach (*P* = 0.03), which was subsequently investigated by pairwise comparisons using the Mann-Whitney test. This statistically significant difference stemmed from higher levels of SIV-specific CD4+ T-cells in Group 1 compared to Group 2 (*P* = 0.004). Groups 1, 2, 3, and 4 are color coded in pink, green, beige, and black, respectively. Lines represent medians, and each symbol corresponds to one vaccinee. N.T., not tested.

We also analyzed vaccine-induced SIV-specific T-cell responses in peripheral blood from macaques in Groups 1–4 by ICS at the time of the first IR SIV challenge. Except for a few high responders, the vast majority of animals had low or undetectable CD4+ T-cell responses (Fig 3). Interestingly, while the limited set of antigens delivered to Group 4 resulted in robust but narrowly focused CD8+ T-cell responses in some of the monkeys, increasing the number of immunogens delivered to Groups 1–3 decreased the frequency of CD8+ T-cells recognizing each viral protein (Fig 3A–3D). Despite these differences, the total magnitude of SIV-specific CD8+ T-cell responses elicited in Groups 1–4 was equivalent (Fig 3E), suggesting that there are constraints to the induction of high frequency, broadly targeted T-cell responses by vaccination. Vaccine-induced SIV-specific CD4+ T-cells were also similar among the groups, except for slightly higher levels of these responses in Group 1 compared to Group 2 (Fig 3E).

**Fig 4:**
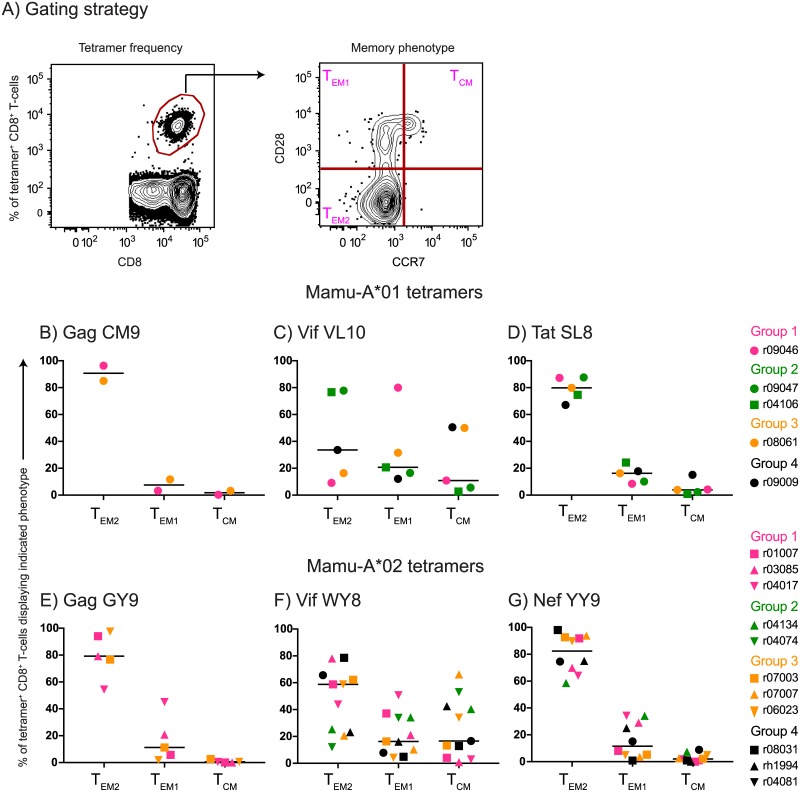
Memory phenotype of vaccine-induced SIV-specific CD8+ T-cells in PBMC at the time of SIV challenge. (A) Gating strategy used to delineate fully differentiated effector memory (T_EM2_), transitional memory (T_EM1_), or central memory (T_CM_) subsets within tetramer+ CD8+ T-cells in peripheral blood. Mamu-A*01 tetramers folded with the following peptides were analyzed: Gag CM9 (B), Vif VL10 (C), and Tat SL8 (D). Mamu-A*02 tetramers folded with the following peptides were analyzed: Gag GY9 (E), Vif WY8 (F), and Nef YY9 (G). Groups 1, 2, 3, and 4 are color coded in pink, green, beige, and black, respectively. Lines represent medians, and each symbol corresponds to one vaccinee.

Heterologous PBB regimens in mice have been shown to induce high frequencies of CD8+ T_EM_ that recirculate through extra-lymphoid anatomical sites [30, 31]. Although we did not determine the frequency of vaccine-elicited CD8+ T-cells in effector tissues in the present study, we characterized the memory phenotype of tetramer+ CD8+ T-cells in blood at the time of the first IR SIV challenge. We delineated tetramer+ CD8+ T-cells as T_CM_, transitional memory (T_EM1_), or fully differentiated T_EM_ (T_EM2_) based on their expression pattern of CD28 and CCR7 (Fig 4A) [42]. Curiously, while the majority of vaccine-induced CD8+ T-cells targeting epitopes in Gag, Tat, and Nef exhibited a T_EM2_ signature, there was great variability in the proportion of Vif-specific CD8+ T-cells displaying this phenotype (Fig 4B–4G).

**Fig 5:**
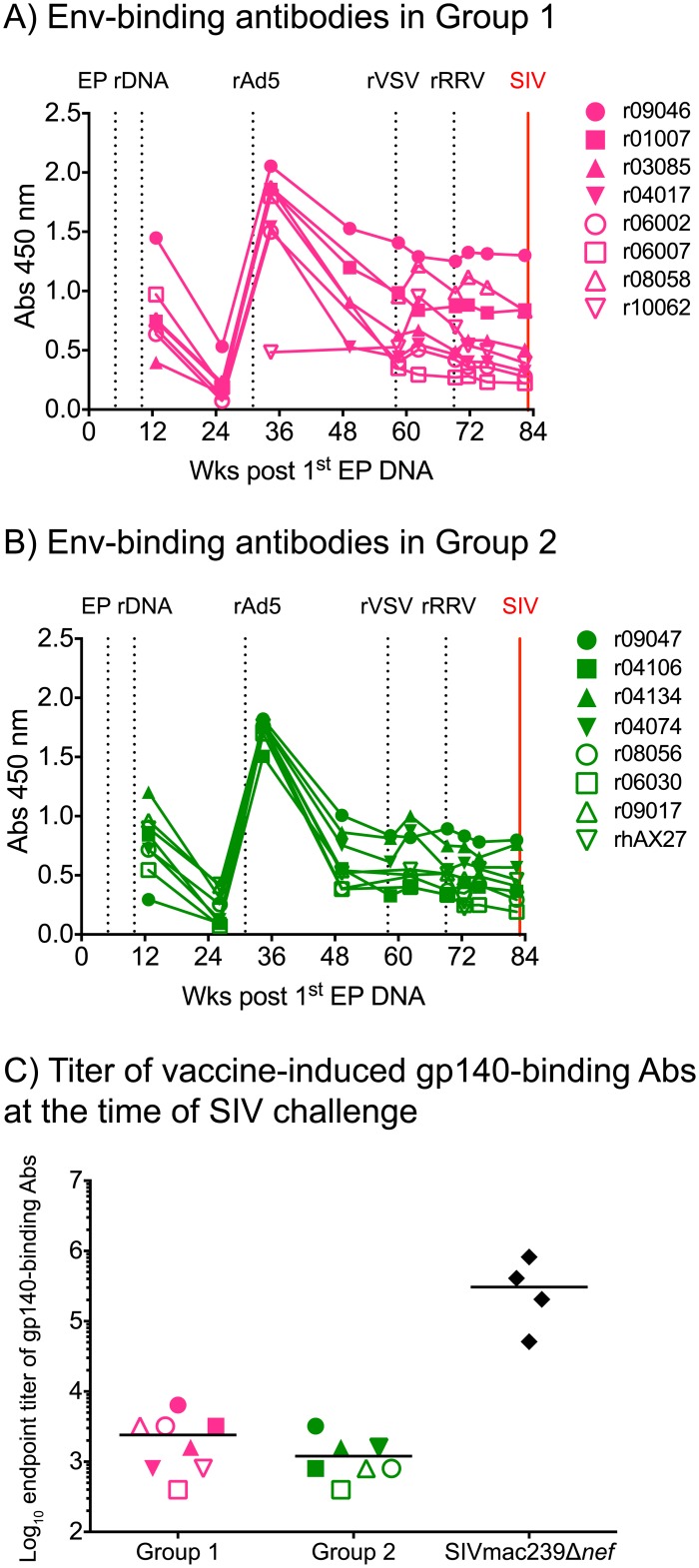
Vaccine-induced Env-binding antibodies in Groups 1 and 2. Binding antibodies to Env were measured by ELISA using plate-bound gp140 at multiple time points during the vaccine phase. Straight 1:200 dilutions of plasma from each of the vaccinees in Group 1 (A) and Group 2 (B) were used for this analysis. Monkey r10062 was not primed with EP rDNA since it was enrolled in Group 1 shortly before the rAd5 boost as a replacement for a rhesus macaque that died unexpectedly. C) Log-transformed endpoint titers of vaccine-induced Env-binding antibodies in Groups 1 and 2 at the time of SIV challenge. As a reference, these values were plotted alongside the endpoint titers of Env-binding antibodies in four rhesus macaques that had been infected with SIVmac239Δ*nef* for 28 weeks as part of a previous experiment (43).

We monitored the levels of vaccine-induced gp140-binding IgG antibodies in plasma from the Group 1 and Group 2 animals throughout the vaccine phase by semi-quantitative ELISA. Except for r10062 in Group 1, all animals already had detectable Env-specific antibodies after the third EP rDNA immunization (Fig 5A & 5B). Monkey r10062 was not primed with EP rDNA since it was enrolled in Group 1 shortly before the rAd5 boost as a replacement for a rhesus macaque that died unexpectedly. Anti-Env humoral responses underwent a sharp but transient increase after the rAd5 boost in all animals (but r10062) in Groups 1 and 2, and subsequently plateaued at levels that remained stable until the time of challenge (Fig 5A & 5B). Neither the rVSV nor the rRRV vaccinations significantly boosted anti-gp140 antibody levels (Fig 5A & 5B). The endpoint titers of vaccine-elicited gp140-binding antibodies in Groups 1 and 2 at the time of the first IR SIV exposure ranged from 400 to 6,400 (Fig 5C). As a reference, these levels were nearly two logs lower than those measured in monkeys that had been infected with SIVmac239Δ*nef* for 28 wks as part of a previous experiment conducted by our group [43].

**Fig 6:**
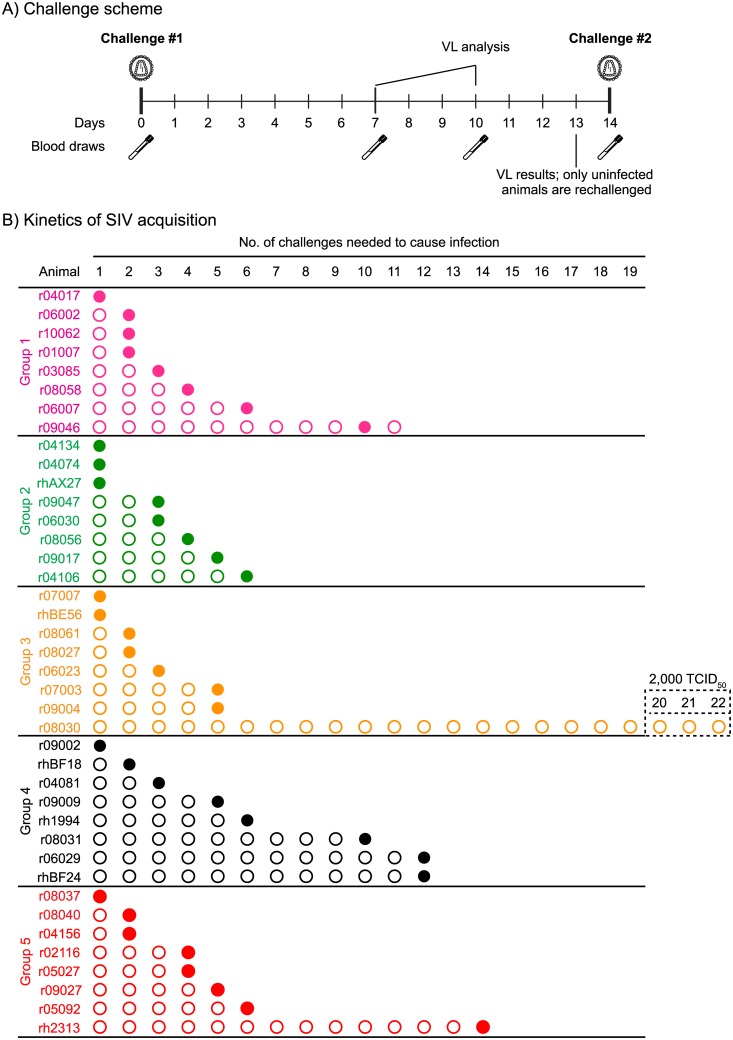
Outcome of repeated marginal dose SIVmac239 IR challenges. A) Challenge scheme. Macaques were exposed to SIV on day 0 and subsequently bled on days 7 and 10. Plasma collected at these two occasions was assayed for the presence of SIV RNA and a decision was made as to whether or not challenge the animals on day 14. Macaques that remained aviremic on both days 7 and 10 were re-challenged, whereas monkeys with a positive VL on either of these days were not re-challenged. For logistical reasons, vaccinees in Groups 1 and 2, as well as four monkeys in Group 5 (r04156, r02116, r05027, and r09027) were challenged for the first time at wk 14 post rRRV. The Group 3 and Group 4 vaccinees, as well as the remaining monkeys in Group 5 (r08037, r08040, r05092, and rh2313) were challenged for the first time at wk 20 post rRRV. B) Kinetics of SIVmac239 acquisition in macaques in Groups 1–5. Individual animals in each of Groups 1–4 are depicted along with the challenge that infected them (filled circles). Animal r09046 was challenged 11 times because it was aviremic on days 7 and 10 following the 10^th^ SIV exposure. However, a subsequent analysis revealed that this animal was viremic on day 14 post challenge #10 (i.e., also the day of the 11^th^ SIV challenge), indicating that it likely acquired SIV infection after the 10^th^ exposure. This is depicted in panel B as an additional empty circle (non-infecting exposure) placed in front of r09046 at challenge #11. Animal r08030 in Group 3 resisted 22 IR challenges with SIVmac239 (the last three at 2,000 TCID_50_).

To assess the efficacy of the various combinations of SIV immunogens delivered to Groups 1–4 by the rDNA/rAd5/rVSV/rRRV regimen, all vaccinees and the control animals in Group 5 were subjected to repeated IR challenges with a marginal dose (200 TCID_50_) of SIVmac239. For logistical reasons, the 40 macaques in the present experiment were staggered in two challenge cohorts. Groups 1, 2, and half of Group 5 (cohort #1) were challenged first at wk 83 after the first EP rDNA vaccination (Fig 1). Groups 3, 4, and the other half of Group 5 (cohort #2) were challenged at wk 89 post initiation of the vaccine regimen (Fig 1). Of note, the total magnitude of vaccine-induced SIV-specific T-cell responses was not significantly different between the groups challenged at either wk 83 or 89 after the first EP rDNA vaccination (Fig 3E). In both cohorts, macaques were exposed intrarectally to SIV every other week, and VLs were determined in plasma samples collected on days 7 and 10 after each challenge (Fig 6A). If a monkey was aviremic on both occasions, it was challenged again on day 14, thereby initiating a new cycle of challenges. However, in case of a positive VL on either day 7 or day 10, the animal was not re-challenged and its VLs were monitored until wk 20 post infection (PI). This strategy enabled us to identify the infecting exposure for all animals in this experiment, except for r09046, in which the first positive VL was detected on day 14 after the 10^th^ SIV exposure. As a result, this animal ended up being challenged eleven times (Fig 6B), even though it likely acquired SIV infection after the 10^th^ exposure.

**Fig 7:**
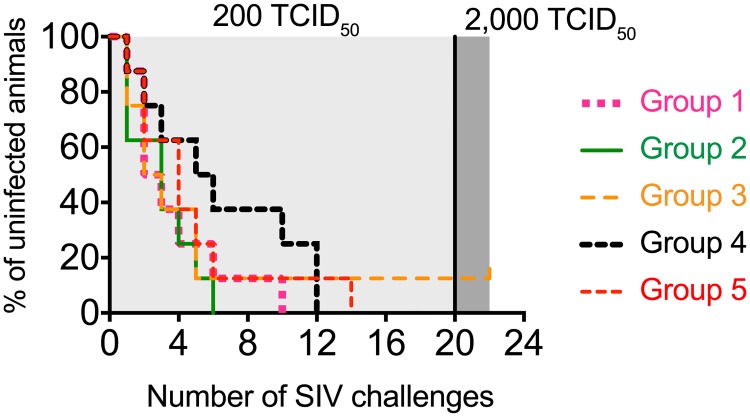
Kaplan-Meier rate of infection after repeated IR challenges with SIVmac239. Macaques in Groups 1–5 were inoculated intrarectally with 200 TCID_50_ of SIVmac239 every other week (light shade of gray). Since the Group 6 vaccinee r08030 remained uninfected after 19 challenges with 200 TCID_50_, the dose of the inoculum was increased 10-fold (2,000 TCID_50_) in the three subsequent exposures (dark shade of gray). Macaque r08030 still remained uninfected after these three high-dose SIV challenges and was not re-challenged. The rate of SIV infection in Groups 1–4 was not significantly different than that of Group 5 (*P* > 0.34).

Surprisingly, the rate of SIV acquisition in Group 4 appeared delayed compared to the other groups (Figs 6B & 7). Indeed, while all of the animals in Group 2 and all but one of the monkeys in Groups 1, 3, and 5 became infected by the 6^th^ SIV exposure, three vaccinees in Group 4 (r08031, r06029, and rhBF24) were still uninfected after six challenges (Fig 6B). These animals remained aviremic after the 7^th^, 8^th^, and 9^th^ SIV exposures (Fig 6B). They also had no detectable T-cell responses against SIV Ag that were not included in the vaccine after the 8^th^ IR challenge (S1 Fig). Monkey r08031 eventually acquired infection after the 10^th^ challenge while both r06029 and rhBF24 became infected after the 12^th^ exposure (Fig 6B). Despite the slower kinetics of SIV acquisition in these three vaccinees, neither Group 4 nor any of the other vaccinated groups differed in a statistically significant fashion from the control group in their rates of infection (Fig 7).

Two animals in the present experiment were unusually resistant to SIV infection. The Group 5 control monkey rh2313 resisted 13 IR challenges before becoming infected after the 14^th^ exposure (Fig 6B). Notably, the Group 3 vaccinee r08030 remained uninfected after 22 IR challenges with SIVmac239, of which the last three exposures delivered a ten-fold higher inoculum (2,000 TCID_50_) (Fig 6B). Previous studies have reported that expression of certain combinations of *TRIM5* alleles, particularly *TRIM5*^*TFP/CypA*^ and to a lesser extent *TRIM5*^*TFP/TFP*^, can affect susceptibility to rectal infection with SIVsmE660 [44, 45]. While monkeys rh2313 and r08030 had moderately restrictive (*TRIM5*^*TFP/Q*^) and restrictive (*TRIM5*^*TFP/TFP*^) genotypes (Table 1), respectively, it is hard to conclude that *TRIM5* allele combinations influenced the rate of SIV infection in this experiment since SIVmac239 has been shown to be refractory to TRIM5α restriction [46]. Additionally, we have previously shown that expression of restrictive *TRIM5* alleles was not associated with delayed SIVmac239 infection in rectally challenged rhesus macaques [45]. Thus, it is not clear why r08030 and rh2313 resisted more challenges than the remaining monkeys in this experiment.

**Fig 8:**
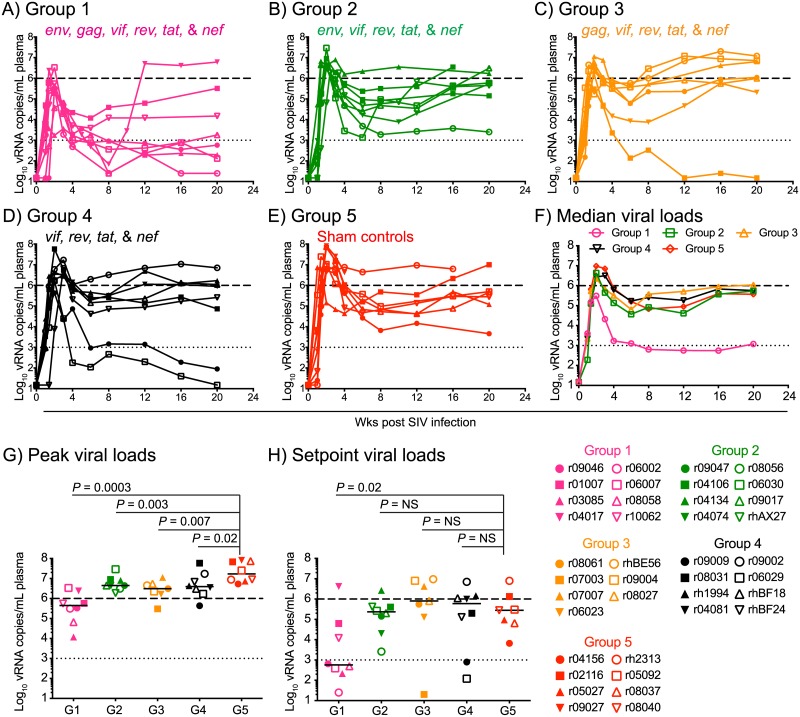
Plasma virus concentrations after SIVmac239 infection. Viral load (VL) traces for individual animals in Group 1 (A), Group 2 (B), Group 3 (C), Group 4 (D), and Group 5 (E). F) Median VLs for Groups 1–5. The median peak (G) and setpoint (H) VLs of Groups 1–4 were compared to those of Group 5 using the Mann-Whitney test. VLs were log-transformed and correspond to the number of vRNA copies/mL of plasma. The dotted lines in all the graphs are for reference only and indicate a VL of 10^3^ vRNA copies/mL. The dashed lines are also for reference only and denote a VL of 10^6^ vRNA copies/mL. Groups 1, 2, 3, 4, and 5 are color coded in pink, green, beige, black, and red, respectively. Lines represent medians and each symbol corresponds to one vaccinee.

Importantly, 5/8 Group 1 vaccinees controlled viral replication to <2,000 vRNA copies/mL of plasma in the chronic phase (Fig 8A & 8H). Although the Group 1 monkey r04017 manifested post peak control of viremia until wk 8 PI, SIV replication surged shortly afterwards, perhaps due to viral escape (Fig 8A). Group 1 exhibited significantly lower peak VLs than the control group, and was the only vaccinated group to reduce chronic phase VLs to a statistically significant level (Fig 8G & 8H).

Interestingly, the virologic control manifested by the Group 1 vaccinees was largely abrogated by the removal of *gag* (Group 2), *env* (Group 3), and both *gag* and *env* (Group 4) from the set of vaccine-encoded immunogens. Indeed, while Groups 2–4 experienced modest, yet statistically significant, reductions in peak viremia, none of these vaccinated groups significantly decreased chronic phase VLs (Fig 8G & 8H). Of note, one monkey in Group 3 (r07003) and two monkeys in Group 4 (r09009 and r06029) controlled viral replication to <1,000 vRNA copies/mL in the chronic phase (Fig 8C & 8D), although it is not clear why these animals fared better after infection than their group counterparts. Together, these results suggest that vaccine-induced immune responses targeting both Gag and Env were crucial for the virologic containment manifested by the Group 1 vaccinees.

**Fig 9:**
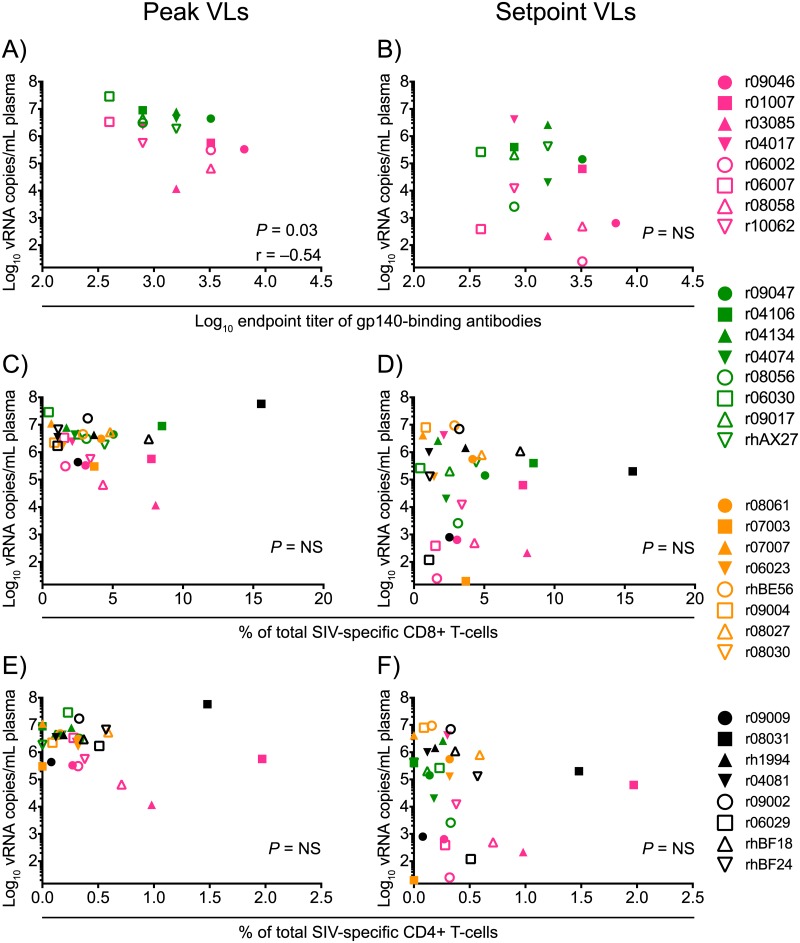
Immune correlates analysis of virologic control of SIVmac239 replication. Three vaccine-induced immune parameters were used for this analysis: the log-transformed titers of gp140-binding antibodies in Groups 1 and 2 at the time of SIV challenge (A and B); the total frequency of SIV-specific CD8+ T-cell responses in Groups 1–4 at the time of SIV challenge (C and D); and the total of frequency of SIV-specific CD4+ T-cell responses in Groups 1–4 at the time of SIV challenge (E and F). These variables were compared with each animal’s peak (A, C, and E) or setpoint (B, D, and F) VLs using the Spearman rank correlation test. Groups 1, 2, 3, and 4 are color coded in pink, green, beige, and black, respectively, and each symbol denotes one monkey.

**Fig 10:**
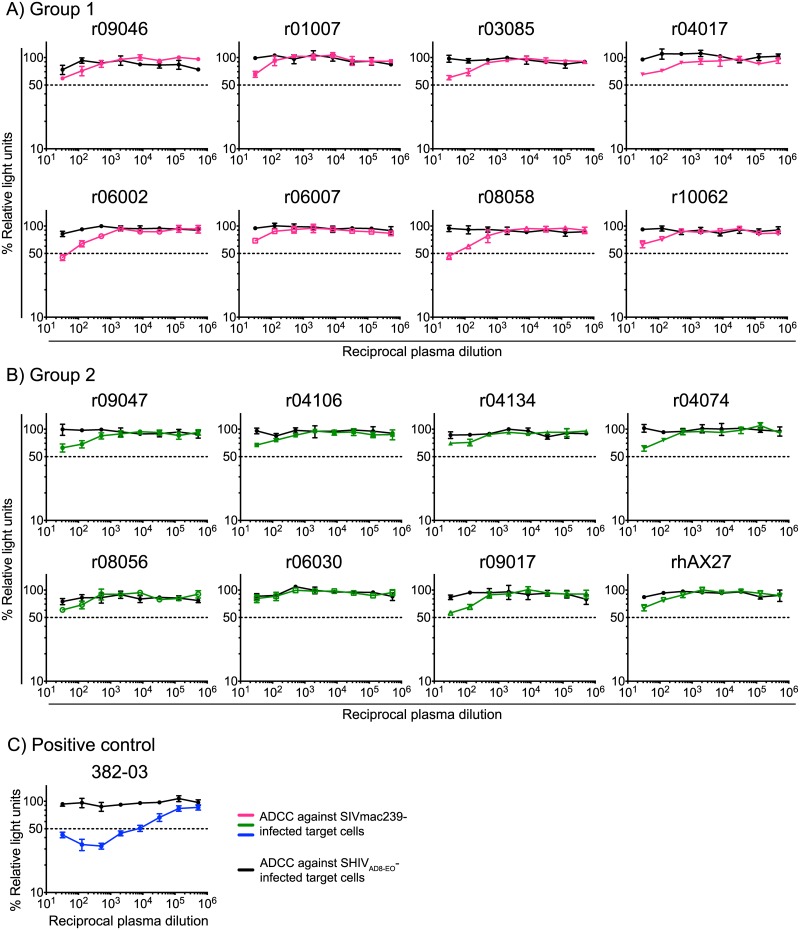
ADCC titers in vaccinated macaques in Groups 1 and 2. Plasma collected from vaccinated macaques in Groups 1 (A) and 2 (B) at the time of the first SIV challenge was screened for ADCC activity against SIVmac239-infected target cells (pink, green, and blue lines). SHIV_AD8-E0_-infected cells were used as internal controls for non-specific killing (black lines). The decrease in relative light units indicates the loss of virus-infected cells in the presence of an NK cell line during the duration of the assay. Dashed lines denote 50% activity. C) Plasma from an SIV-infected rhesus macaque (382–03) with a defined ADCC titer against SIVmac239-infected cells was used as a positive control for these measurements.

Lastly, we investigated potential mechanisms for the differential control of viral replication manifested by the vaccinees in Groups 1–4. This analysis revealed that high titers of vaccine-induced gp140-binding antibodies at the time of challenge correlated with lower peak VLs in Groups 1 and 2 (Fig 9A). However, these antibody responses did not predict control of chronic phase viremia (Fig 9B). Despite the association with peak VLs, none of the animals exhibited serological neutralizing activity against SIVmac239 at the time of challenge (S2 Fig). Since antibody dependent cellular cytotoxicity (ADCC) has been linked to the protective efficacy of live-attenuated SIV vaccination [47], we also measured this parameter in plasma from the Group 1 and Group 2 vaccinees. This analysis revealed little or no ADCC activity against SIVmac239-infected target cells at the time of the first SIV exposure (Fig 10). We also examined the predictive value of the total magnitude of vaccine-elicited SIV-specific CD4+ or CD8+ T-cell responses in Groups 1–4 and found no correlation between these variables and suppression of SIV replication (Fig 9C–9F). Collectively, these data suggest that virologic control of the highly pathogenic SIVmac239 clone might be achieved by vaccine regimens that elicit high titers of gp140-binding antibodies and T-cells targeting multiple viral proteins (Env and Gag inclusive).

## Discussion

Here we conducted a head-to-head comparison of the immunogenicity and protective efficacy of four different sets of SIV inserts delivered by a novel PBBB regimen. All four sets included the regulatory and accessory proteins Vif, Rev, Tat, and Nef, which were administered by themselves (Group 4) or with the addition of Gag (Group 3), Env (Group 2), or both Gag and Env (Group 1). Interestingly, despite differences in the number and size of vaccine-encoded immunogens, the total magnitude of SIV-specific CD8+ T-cells elicited by vaccination was equivalent in Groups 1–4. This was explained by some of the Group 4 vaccinees mounting robust CD8+ T-cell responses focused almost entirely on Vif and/or Nef. Although responses against these proteins were also detected in Groups 1–3, they tended to be lower in frequency, possibly reflecting a diversion of the SIV-specific T-cell response toward the larger Env and Gag proteins. These results are consistent with those reported by Hel *et al*. in the context of SIV vaccination and suggest that, at least as reflected in peripheral blood, total vaccine-elicited Ag-specific CD8+ T-cells are poised to proliferate until a “ceiling” level is reached, regardless of the number or size of inserts delivered by vaccination [48]. It is not clear how this ceiling of memory CD8+ T-cell expansion is regulated, but host-intrinsic factors such as T-cell competition for Ag-bearing dendritic cells, immunological experience, naïve T-cell precursor frequencies, and CD8+ T-cell immunodominance might be involved. Collectively, these results underscore the difficulty of engendering HIV-1-specific CD8+ T-cell responses by vaccination that are both large in size and broad in epitope recognition.

Importantly, increasing the number of immunogens delivered by vaccination appeared to enhance control of SIVmac239 replication. Indeed, Group 1 exhibited the greatest reduction in peak VLs, with 5/8 vaccinees in this group going on to control chronic phase viremia to less than 2,000 vRNA copies/mL. In contrast, Groups 2–4 manifested only modest suppression of peak VLs and no reduction in chronic phase VLs. The improved virologic control manifested by the Group 1 vaccinees might have been due to a synergy between vaccine-induced T-cells targeting epitopes in multiple viral proteins and anti-Env humoral responses. The latter possibility is supported by the inverse correlation between titers of vaccine-induced Env-binding antibodies and control of acute phase viremia (Fig 9), even though these antibodies could not neutralize SIVmac239 *in vitro* (S2 Fig). ADCC was detectable in several animals but only at high concentrations of plasma (Fig 10). It is worth mentioning that we did not evaluate antibody functions at the site of virus exposure. Since peripheral blood is an imperfect proxy for the mucosal milieu, additional characterizations of Env-specific antibodies in rectal secretions could have shed light into the mechanisms by which macaques in Groups 1 and 2 suppressed acute phase viremia. Since HIV-1 is transmitted primarily via unprotected intercourse, a successful vaccine may need to induce antiviral immunity at the reproductive and gastrointestinal tracts. Thus, pre-clinical evaluations of HIV-1 vaccine regimens should include mucosal samplings of relevant sites of virus transmission.

Our data also suggest that control of lentivirus replication might be facilitated by vaccine-induced immune responses targeting both Gag and Env since the absence of either (Groups 2 and 3) or both of these responses (Group 4) largely abrogated the virologic control afforded by the Group 1 regimen. Of note, since we did not evaluate the protective efficacy of a *gag* and *env-*only PBBB regimen, it is formally possible that vaccine-induced immune responses against Gag and Env were sufficient for the superior performance of Group 1. Indeed, the utility of Gag and Env as vaccine immunogens has been demonstrated by several previous studies [6, 36, 49, 50]. Nevertheless, we favor the interpretation that vaccine-elicited immune responses against Gag and Env acted in synergy with those targeting Vif, Rev, Tat, and Nef to control viral replication in Group 1. In support of this view, macaques vaccinated with rDNA/rNYVAC encoding SIV *gag*, *pol*, *env*, and a *rev-tat-nef* fusion insert manifested better control of SIVmac251 replication than did recipients of the same regimen lacking nonstructural SIV genes [48]. Furthermore, the efficacy of rAd5-based immunization protocols against IR SIV challenges appears to increase with the progressive incorporation of inserts encoding viral proteins [49, 51–55]. Thus, our data and those from others suggest that vaccine-mediated control of lentivirus replication might be improved by inducing immune responses against Gag, Env, and the remaining viral proteins as well.

The fact that vaccinees in Groups 1 and 2 became infected at the same rate as the control group contrasts with recent studies reporting that vaccine-induced Env-binding antibodies can affect the rate of SIVmac251 acquisition after IR challenge [6, 8, 9, 56]. While it is difficult to compare these discrepant outcomes–considering the distinct underlying experimental designs, the stringency of SIVmac239 as a challenge virus may explain these different results. Despite being genetically related, SIVmac239 and the SIVmac251 isolates used in the studies cited above differ in important ways. For instance, while SIVmac239 is a molecular clone, resulting in little sequence variability among different stocks, SIVmac251 consists of a swarm of viral quasispecies. Indeed, Del Prete *et al*. have recently reported considerable sequence diversity among different stocks of SIVmac251 [57]. Although this feature of SIVmac251 might be useful for tracking the number of transmitted/founder viruses in mucosal challenge studies, it can also result in unwanted variability. For example, there are conflicting reports on the susceptibility of SIVmac251 to TRIM5α restriction [58–60]. The passage history of SIVmac251 stocks has also been shown to impact their susceptibility to antibody neutralization *in vitro* [61], and SIVmac251 *env* clones displaying tier 1 and tier 3 neutralization profiles have been isolated [14]. The SIVmac239 Env, on the other hand, is known to be consistently resistant to antibody neutralization [11–13, 15]. Similar to HIV-1 Env immunogens, vaccination with SIVmac239 Env elicits primarily tier 1 nAbs and, to our knowledge, no vaccine regimen has been able to consistently engender potent nAbs against SIVmac239 [14]. Ultimately, it will be important to determine if these differences have any translational relevance, especially since vaccine efficacy against SIVmac251 has been recently used to justify a phase 2b/3 efficacy trial of an ALVAC-HIV/gp120 vaccine regimen in South Africa [62].

Six animals in the present experiment resisted multiple IR challenges with SIVmac239: one in Group 1 (r09046), one in Group 3 (r08030), three in Group 4 (r08031, r06029, and rhBF24), and one in the control group (rh2313). It is not clear why these monkeys exhibited this phenotype. Of these monkeys, only r09046 expressed a combination of *TRIM5* alleles that has been significantly associated with resistance to rectal SIVsmE660 infection (*TRIM5*^*TFP/CypA*^) [46], although this effect was not observed in macaques following IR challenges with SIVmac239 [45]. The remaining animals were positive for either moderately restrictive or susceptible *TRIM5* allele combinations (Table 1), indicating that TRIM5α restriction of SIV infection cannot solely account of these animals’ resistance to SIV acquisition. It is also noteworthy that mucosal transmission of immunodeficiency viruses can be shaped by both selective and stochastic events, and factors such as mucosal integrity, local inflammation, and the amount of intraluminal feces at the time of virus exposure have been proposed to influence susceptibility to rectal lentivirus infection [63–65]. In this regard, we cannot rule out the possibility that the delayed infection rate observed in the aforementioned animals was due to chance. Curiously, however, three of these six macaques were in Group 4, the most distinctive immunological feature of which was the development of CD8+ T-cell responses focused on Vif and Nef. These results are similar to those observed in another SIV vaccine trial recently conducted by our laboratory. The macaques in this other study expressed the elite control-associated MHC-I allele Mamu-B*08 and were vaccinated with a rAd5/rVSV/rRRV PBB regimen expressing *vif*, *rev*, *tat*, and *nef* inserts matching the SIVmac239 challenge virus. Similar to the immunization protocol presented here, the rRRV vectors were delivered to these Mamu-B*08+ vaccinees via both the IV and IR routes. These animals also mounted CD8+ T-cell responses predominantly focused on Vif and Nef since Mamu-B*08 restricts immunodominant epitopes in these proteins [66]. Notably, after six IR challenges with the same dose and stock of SIVmac239 utilized here, 4/10 Mamu-B*08+ vaccinees remained uninfected whereas all MHC-I-matched control monkeys became infected. In keeping with these outcomes, Xu *et al*. have recently assessed the efficacy of a mucosal T-cell-based vaccine encoding SIV accessory proteins in rhesus macaques against repeated IR challenges with SIVmac251 [67]. Tellingly, 3/6 vaccinees versus 1/6 controls remained aviremic after five IR exposures with a marginal dose of SIVmac251. The small sample sizes of these three independent experiments obviously preclude any definitive conclusions. However, if these similar challenge outcomes are considered together, they suggest that vaccine-induced CD8+ T-cells against accessory proteins may have some capacity to prevent systemic infection in the absence of anti-Env antibodies.

How could vaccine-elicited SIV-specific CD8+ T-cells mediate such an effect since CD8+ T-lymphocytes can only eliminate viruses after co-localizing with cells that are already infected? It is possible that tissue-resident memory T-cells (T_RM_) induced by vaccination intercepted the initial foci of infected cells in the rectum and/or its associated lymphoid structures before the infection became systemic. T_RM_ resemble T_EM_ in their fast acting antiviral properties [21, 68], but in contrast to T_EM_, T_RM_ do not recirculate and remain permanently positioned in effector tissues [68]. T_RM_ have been the focus of intense research lately since these cells participate in the first line of defense against pathogens. Unfortunately, the logistics of conducting a large monkey experiment precluded us from searching for vaccine-induced SIV-specific T_RM_ prior to the challenge phase. Nevertheless, based on results from previous studies [69, 70], we speculate that the simultaneous delivery of the rVSV and rRRV vectors via the IV and IR routes may have increased the frequency of SIV-specific CD8+ T-cells at relevant sites of virus transmission and amplification. Importantly, mounting evidence suggests that CD8+ T-cells can suppress immunodeficiency virus replication shortly after mucosal transmission and before a long-lived viral reservoir is established. For example, 50% of RhCMV/SIV vaccinees manifest stringent control of viral replication early after mucosal SIVmac239 infection [26]. This outcome may reflect vaccine-induced T-cell-mediated restriction of viral spread beyond a relatively small and short-lived population of initially infected cells, with clearance or spontaneous decay of this population over time [27]. Furthermore, and as mentioned above, a recent monkey study evaluated the efficacy of a T-cell-based SIV vaccine administered both intramuscularly and intrarectally and reported that a fraction of vaccinees remained aviremic after repeated marginal dose IR challenges with SIVmac251 [67]. Collectively, these results lend support to the hypothesis that vaccine-induced T-cells may be able to prevent systemic infection. Ultimately, however, larger and appropriately powered monkey trials will be needed to validate this hypothesis.

Since Gag has largely been the preferred target for the induction of HIV-1-specific cellular immunity by vaccination [16], the Ag specificity of vaccine-elicited CD8+ T-cells in Group 4 merits discussion since they were focused almost entirely on the accessory proteins Vif and Nef. Although the idea of using the HIV-1 accessory and regulatory proteins as vaccine immunogens has been proposed previously [71], relatively few monkey studies have explored the protective efficacy of vaccine-induced T-cell responses against these targets in the face of stringent SIV challenges. We have recently shown that high frequency, Nef-specific CD8+ T-cells generated by a PBB regimen did not protect Mamu-B*08+ macaques from the same IR SIVmac239 challenge employed here [72]. This observation raises the possibility that Vif-specific CD8+ T-cells might have been important for the delayed SIV infection kinetics observed in Group 4 and in the aforementioned Mamu-B*08+ vaccinees. Curiously, a recent analysis of immune responses in a large cohort of HIV-1-exposed seronegative individuals revealed that T-cells, especially those targeting Vif, correlated inversely with infection risk [41]. Furthermore, we and others have reported associations between vaccine-elicited T-cell responses against Vif and control of SIV replication in rhesus macaques [39, 73]. These analyses, however inconclusive, warrant additional investigation into the antiviral role of Vif-specific CD8+ T-cells *in vivo*.

In conclusion, here we show that expanding the number of vaccine-encoded Ag improved control of viral replication in SIVmac239-infected rhesus macaques and that vaccine-induced immune responses against Env and Gag were required for this effect. In this regard, we are currently exploring the efficacy of an enhanced mixed modality immunization regimen encoding the entire SIV proteome against IR challenge with SIVmac239. The tantalizing hint of delayed SIV acquisition in macaques vaccinated with Vif and Nef has also prompted us to begin to evaluate the protective effects of vaccine-induced CD8+ T-cell responses focused on Vif in a large monkey experiment. Together, these results might be relevant for the design of future HIV-1 vaccine regimens since they provide clues as to the most effective targets of anti-lentivirus immunity.

## Materials and methods

### Research animals and ethics statement

The details regarding animal welfare described herein are either similar or identical to those published in one of our previous experiments [74]. “The Indian rhesus macaques (*Macaca mulatta*) utilized in this study were housed at the Wisconsin National Primate Research Center (WNPRC). All animals were cared for in accordance with the guidelines of the Weatherall report and the principles described in the National Research Council’s Guide for the Care and Use of Laboratory Animals under a protocol approved by the University of Wisconsin Graduate School Animal Care and Use Committee” (animal welfare assurance no. A3368-01; protocol no. G00671 and G005145) [75]. “Furthermore, the macaques in this study were managed according to the animal husbandry program of the WNPRC, which aims at providing consistent and excellent care to nonhuman primates at the center. This program is employed by the Colony Management Unit and is based on the laws, regulations, and guidelines promulgated by the United States Department of Agriculture (e.g., the Animal Welfare Act and its regulations, and the Animal Care Policy Manual), Institute for Laboratory Animal Research (e.g., Guide for the Care and Use of Laboratory Animals, 8^th^ edition), Public Health Service, National Research Council, Centers for Disease Control, and the Association for Assessment and Accreditation of Laboratory Animal Care International. The nutritional plan utilized by the WNPRC is based on recommendations published by the National Research Council. Specifically, macaques were fed twice daily with 2050 Teklad Global 20% Protein Primate Diet and food intake was closely monitored by Animal Research Technicians. This diet was also supplemented with a variety of fruits, vegetables, and other edible objects as part of the environmental enrichment program established by the Behavioral Management Unit. Paired/grouped animals exhibiting stereotypical and/or incompatible behaviors were reported to the Behavioral Management staff and managed accordingly. All primary enclosures (i.e., stationary cages, mobile racks, and pens) and animal rooms were cleaned daily with water and sanitized at least once every two weeks.” Lights were on a 12:12 diurnal schedule. Vaccinations were performed under anesthesia (Ketamine administered at 5–12 mg/kg depending on the animal) and all efforts were made to minimize suffering. Euthanasia was performed at the end of the study or whenever an animal experienced conditions deemed distressful by one of the veterinarians at the WNPRC. All euthanasia were performed in accordance with the recommendations of the Panel on Euthanasia of the American Veterinary Medical Association and consisted of an IV overdose (greater than or equal to 50 mg/kg or to effect) of sodium pentobarbital or equivalent, as approved by a clinical veterinarian, preceded by ketamine (at least 15 mg/kg body weight) given by the intramuscular (IM) route. Additional animal information, including MHC-I and *TRIM5* alleles, age at the beginning of study, and sex, is shown in Table 1.

### Vaccinations

Seven rDNA constructs consisting of different versions of the pCMVkan plasmid were used in this experiment [55]. Six of them expressed one of the following codon-optimized SIVmac239 inserts: *env* (full length gp160), *gag*, *vif*, *rev-tat*, and *nef*. The seventh rDNA construct consisted of “empty” pCMVkan lacking any insert and was administered to the control animals in Group 5. The macaques in Groups 1–5 were vaccinated intramuscularly with a mixture of 1.0 mg of the appropriate rDNA plasmid (see Fig 1) and 0.1 mg of the IL-12-expressing AG157 plasmid using the TriGrid *in vivo* electroporation system (Ichor Medical Systems, Inc., San Diego, CA) [76]. Macaques in Groups 1–5 were primed with EP rDNA three times at 5-wk intervals. Muscles in the thighs and forearms were used for these vaccinations and these anatomical sites were rotated in subsequent immunizations so that each location did not receive vectors encoding the same SIV inserts twice.

The rAd5 boost occurred at wk 31 following the first EP rDNA immunization. Five rAd5 vectors were used in this experiment [55, 77]. Four of these rAd5 vectors expressed one of the following codon-optimized SIVmac239 inserts: *env* (full length gp160), *gag*, a *vif* minigene encoding Vif amino acids (aa) 1–110, and a *nef* minigene encoding Nef aa 45–210. The latter two vectors were produced by Viraquest, Inc, whereas the rest of the rAd5 constructs were produced by the International AIDS Vaccine Initiative. The fifth vector consisted of “empty” rAd5 lacking any insert and was administered to the control animals in Group 5. The appropriate rAd5 vectors were administered intramuscularly to the monkeys in Groups 1–5, with 10^11^ viral particles of each vector delivered to the same sites used for the EP rDNA vaccinations.

The rVSV boost occurred at wk 58 following the first EP rDNA immunization. Six rVSV vectors expressing SIVmac239 inserts were used in this experiment. Five were based on a VSV Indiana vector in which the G gene was repositioned to the 5’ terminus of the genome [78]. By placing the G gene in the most distal gene position relative to the promoter (6^th^ position), its expression was modestly downregulated. The vector was also modified by replacing the VSV Indiana G sequence with that from VSV New Jersey. The five VSV constructs based on this vector included: 1) rVSV-EnvΔct_4_-G_nj6_ encoded SIV Env protein with a cytoplasmic tail that was truncated by 159 aa at its carboxyl terminus. This modification increased Env surface expression and genetic stability of rVSV-Env4-G_nj6_-infected cells *in vivo* [78, 79]. The *envΔct*_*4*_ gene was inserted into the 4^th^ position relative to the 3’ end of the VSV genome. 2) rVSV-Gag_1_-G_nj6_ encoded the full-length Gag polyprotein. The *gag* gene was inserted into position 1 of the VSV genome. 3) rVSV-Nef_1_-G_nj6_ encoded a Nef protein lacking its myristoylation signal. This myristoylation-deficient *nef* gene was inserted into position 1 of the VSV genome. 4) rVSV-TatRev_1_-G_nj6_ encoded a fusion of Tat and Rev regulatory proteins. The *tatrev* fusion gene was inserted into position 1 of the VSV genome. 5) rVSV-Vif(mut4)3-G_nj6_ encoded a truncated Vif protein lacking aa 2–41. This Vif vector was developed after finding that the initial rVSV-G_nj6_ constructs expressing full-length *vif* were genetically unstable. To identify a stable Vif vector, we subsequently screened several new vectors containing *vif* inserts with or without coding sequence for aa 2–41, some of which also were modified by shuffling Vif domains. The *vif(mut4)3* gene, which encoded Vif lacking aa 2–41, was among the modified inserts tested that was genetically stable when inserted into position 3 of the VSV genome. Recombinant VSV-G6 vectors were rescued from DNA using a helper-virus free method described earlier [78]. Vaccine vectors also were amplified and purified as described earlier [78]. Although rVSV-Vif(mut4)3-G_nj6_ was genetically stable, infected Vero cells (Meridian Life Science) produced relatively low levels of Vif protein. This caveat prompted us to generate an additional *vif-*expressing rVSV vector using a different vector design. The SIVmac239 *vif* gene was amplified by PCR for insertion in the 5^th^ genomic position between the XhoI and NheI sites in the VSV Indiana genomic clone described by Schnell and collaborators [80]. The *vif* sequence was also modified to include a C-terminal V5 epitope tag for detection by Western blotting. Molecular cloning and rescuing of this rVSV-Vif vector was performed as described before using 293T cell monolayers (ATCC) and recombinant vaccinia virus expressing T7 RNA polymerase [81]. Clonal isolates were prepared by plaque purification using baby hamster kidney (BHK) cells (generously provided by M.A. Whitt). Vif expression was confirmed by infecting 293T cells and analyzing infected cell lysates by Western blotting. A rVSV-Vif plaque isolate was then amplified by infecting 293T cells and subsequently harvesting virus from medium supernatant. This virus-containing medium was passed through a 0.2-μM vacuum filter and then purified by centrifugation (27,000 RPM/90 min/4°C) though a low-density cushion of 10% Optiprep (Sigma). The virus pellet was homogenized in PBS and its titer was determined by plaque assay using BHK cells.

While generating rVSV-Vif, we did not observe the genetic instability described above for the different VSV-Vif-G_nj6_ vectors. We suspect this may be due to full-length Vif expression having an inhibitory effect on VSV replication specifically in Vero cells, which are derived from African Green monkey kidneys [82]. This explanation is based on several observations: 1) rVSV-Vif was generated using human 293T cells and BHK cells and not Vero cells; Vif expression by rVSV-Vif was considerably higher in infected BHK or 293T cells compared to Vero cells; and 3) rVSV-Vif-G_nj6_ vectors were developed by a process based entirely on Vero cells.

The appropriate rVSV vectors for each group were simultaneously delivered via the IV and IR routes. The control animals in Group 5 were vaccinated with “empty” rVSV-G_nj6_. A total dose of 10^8^ PFU of each vector was administered per animal; half was delivered intravenously while the other half was given intrarectally. For both routes, the rVSV vector mixture was administered in 1.0 mL of PBS. All animals in Groups 1–4 were vaccinated with the two *vif-*expressing rVSV vectors described above, rVSV-Vif(mut4)_3_-G_nj6_ and rVSV-Vif.

The rRRV boost occurred at wk 69 following the first EP rDNA immunization. The generation of the rRRV constructs employed here has been described elsewhere [33]. Five rRRV vectors expressing SIVmac239 inserts were used in this experiment. 1) rRRV-SIV-Gag encoded a codon-optimized full-length *gag* gene. 2) rRRV-SIV-wdo-gp160 encoded an *env* gene whose codon usage matched that of the RRV gH gene. 3) rRRV-SIV-RTN3 encoded a fusion of the *rev*, *tat*, and *nef* genes. 4) rRRV-SIV-nef-v5 and 5) rRRV-SIV-vif-v5 each encoded the *nef* or *vif* genes, respectively. Similar to the rVSV vaccinations, the appropriate rRRV vectors for each group were simultaneously delivered via the IV and IR routes. The vaccine formulation for each route consisted of 1.0 mL of PBS containing 7.1×10^7^ genome copies of each rRRV vector. All animals in Groups 1–4 were vaccinated with rRRV-SIV-RTN3, rRRV-SIV-nef-v5, and rRRV-SIV-vif-v5. The control animals in Group 5 were vaccinated with rRRV expressing enhanced fluorescent green protein.

### SIVmac239 challenges

The challenge stock utilized here was produced by the Virology Services Unit of the WNPRC using SIVmac239 hemi-genome plasmids obtained from the NIH AIDS Research and Reference Reagent Program. These plasmids were transfected into 293T cells and the supernatant was propagated on mitogen-activated PBMC from SIV naïve rhesus macaques for several days. The titer of this stock was 90,000 50% tissue culture infective doses (TCID_50_)/mL. Animals in this study were subjected to the IR challenge regimen described in Fig 6. The dose of each exposure was 200 TCID_50_, which corresponded to 4.8×10^5^ viral RNA (vRNA) copies. Plasma VLs were assessed seven and ten days after each exposure. Once an animal had a positive VL, it was no longer challenged.

### SIV viral load measurements

VLs were measured using 0.5 mL of EDTA-anticoagulated rhesus macaque plasma based on a modification of a previously published [83]. Total RNA was extracted from plasma samples using QIAgen DSP virus/pathogen Midi kits, on a QIASymphonyXP laboratory automation instrument platform. Six replicate two step RT-PCR reactions were performed per sample using a random primed reverse transcription reaction, followed by 45 cycles of PCR using the following primers and probe: forward primer: SGAG21: 5’-GTCTGCGTCAT(dP)TGGTGCATTC-3’; reverse primer SGAG22: 5’-CACTAG(dK)TGTCTCTGCACTAT(dP)TGTTTTG-3’; probe: PSGAG23: 5’-FAM-CTTC(dP)TCAGT(dK)TGTTTCACTTTCTCTTCTGCG-BHQ1-3’. The limit of reliable quantitation on an input volume of 0.5 mL of plasma was 15 vRNA copies/mL.

### Memory phenotyping of MHC-I tetramer^+^ CD8^+^ T-cells

Rhesus macaque peripheral blood mononuclear cells (PBMC) were isolated from EDTA blood as described previously [72]. These cells were stained with fluorochrome-labeled MHC-I tetramers obtained from either the NIH Tetramer Core Facility or MBL International Inc. according to a recently published protocol [84]. Up to 800,000 PBMC were incubated with titrated amounts of each tetramer at room temperature (RT) for 45 min and then stained with fluorochrome-labeled monoclonal antibodies (mAbs) directed against the surface molecules CD3 (clone SP34-2), CD8α (clone RPA-T8), CD28 (clone 28.2), CCR7 (clone 150503), CD14 (clone M5E2), CD16 (clone 3G8), and CD20 (clone 2H7). Amine-reactive dye (ARD; Live/DEAD Fixable Aqua Dead Cell Stain; Life Technologies) was also added to this mAb cocktail. After a 25-min incubation at RT, the cells were washed with Wash Buffer (Dulbecco’s PBS with 0.1% bovine serum albumin and 0.45 g/L NaN_3_) and then fixed with PBS containing 2% of paraformaldehyde. The configuration of the Special Order Product BD LSR II cytometer used to acquire the samples and the gating strategy employed to analyze the data have been detailed elsewhere [77]. In sum, we used FlowJo 9.6 to determine the percentages of live CD14^−^CD16^−^CD20^−^CD3^+^CD8^+^tetramer^+^ lymphocytes shown in Fig 2 and to delineate memory subsets within tetramer^+^ populations (Fig 4).

### Intracellular cytokine staining (ICS) assay

PBMC were stimulated with the appropriate pools of SIV peptides in R10 medium (RPMI 1640 medium supplemented with GlutaMax [Life Technologies], 10% FBS [VWR], and 1% antibiotic/antimycotic [VWR]) containing co-stimulatory mAbs against CD28 and CD49d for 9 h at 37°C in a 5.0% CO_2_ incubator. A phycoerythrin-conjugated mAb specific for CD107a was also included in the assay. To inhibit protein transport, Brefeldin A (Biolegend, Inc.) and GolgiStop (BD Biosciences) were added to all tubes 1 h into the incubation period. The antigen stimuli consisted of six pools of peptides (15mers overlapping by 11 aa) spanning (i) the entire Gag polyprotein (aa 1–510), (ii) Env gp120 (aa 1–531), (iii) Env gp41 (aa 516–879), (iv) the entire Vif protein (aa 1–214), (v) the entire Nef protein (aa 1–263), and (vi) both the Rev (aa 1–107) and Tat (aa 1–130) proteins. The final assay concentration of each 15mer was 1.0 μM. We employed the same steps outlined above to stain molecules on the surface of cells and to fix them with 2% of paraformaldehyde. In addition to the same mAbs against CD14, CD16, and CD20 and the ARD reagent described above, the surface staining master mix also included mAbs against CD4 (clone OKT4; Biolegend, Inc.) and CD8 (clone RPA-T8; Biolegend, Inc.). Cells were permeabilized by resuspending them in “Perm Buffer” (1× BD FACS lysing solution 2 [Beckton Dickinson] and 0.05% Tween 20 [Sigma-Aldrich]) for 10 min and subsequently washed with Wash Buffer. Cells were then incubated with mAbs against CD3 (clone SP34-2; BD Biosciences), IFN-γ (clone 4S.B3; Biolegend, Inc.), TNF-α (clone Mab11; BD Biosciences), and CD69 (clone FN50; Biolegend, Inc.) for 1 h in the dark at RT. After this incubation was completed, the cells were washed and subsequently stored at 4°C until acquisition. We analyzed the data by gating first on live CD14–CD16–CD20–CD3+ lymphocytes and then on cells expressing either CD4 or CD8 but not both markers. We then conducted functional analyses within these two compartments. Cells were considered positive for IFN-γ, TNF-α, or CD107a only if they co-expressed these molecules with CD69, a marker of recent activation. Once the appropriate gates were created, we employed the Boolean gate platform to generate a full array of possible combinations, equating to 8 response patterns when testing three functions (2^3^ = 8). Leukocyte activation cocktail (LAC; BD Pharmingen)-stimulated cells stained with fluorochrome-labeled mAbs of the same isotypes as those against IFN-γ, TNF-α, and CD107a guided the identification of positive populations. We used two criteria to determine if responses were positive. First, the frequency of events in each Boolean gate had to be at least 2-fold higher than their corresponding values in background-subtracted negative-control tests. Second, the Boolean gates for each response had to contain ≥10 events. The magnitude of responding CD4+ or CD8+ T-cells shown in Figs 3 and 9 was calculated by adding the frequencies of positive responses producing any combination of IFN-γ, TNF-α, and CD107a. Background subtraction and calculation of the frequencies of responding cells were performed with Microsoft Excel.

### Anti-Env antibody by ELISA

Vaccine induced anti-Env responses were measured by ELISA. To begin, the ELISA plate was coated with 100 μL of purified SIVmac239 gp140 protein (Immune Technology Corp. #IT-001-140p) at a concentration of 0.5 μg/mL and incubated overnight at RT. On the following day, the plate was washed with 1× PBS-Tween20 and wells were blocked with 300 μL of 5% powdered milk in PBS for 1 hr at 37°C. Subsequently, the plate was washed and 100 μL of diluted plasma samples were added to the corresponding wells. After a 1-hr incubation at RT, the plate was washed and 100 μL of a 1:2,000 dilution of Goat Anti-Monkey IgG-HRP antibody (Santa Cruz Biotechnology, sc-2458) were added to all wells for 1 hr at 37°C. Finally, the plate was washed before being developed with 100 μL of 3,3',5,5'-Tetramethylbenzidine (EMD Millipore, 613544-100ML). After a short incubation, the reaction was stopped with TMB Stop Solution (Southern Biotech, 0412–01) and the plate was read (Biotek Synergy 2) at 450 nm. The endpoint antibody titers of vaccine-induced anti-Env antibody responses were measured in serum collected at the time of SIV challenge. These titers were determined as the greatest dilution at which the absorbance in experimental wells was at least two-fold higher than that measured in pooled pre-vaccination serum from all animals in the experiment.

### Antibody dependent cellular cytotoxicity (ADCC) assay

The SIVmac239 and SHIV_AD8-EO_ stocks used in ADCC assays were produced by transfection of infectious molecular clones into HEK293T cells using GenJet transfection reagent (SignaGen). Virus-containing supernatants were collected 48 and 72 hours (h) post-transfection and stored at -80°C. The SHIV_AD8-EO_ clone was provided by Dr. Malcom Martin (NIAID, Bethesda, MD). After heat inactivation for 30 minutes at 56°C, rhesus macaque plasma samples were tested for non-specific ADCC due to the presence of antibodies to human cellular antigens by co-incubating uninfected CEM.NKR-CCR5-sLTR-Luc target cells with an NK cell line (KHYG-1 cells) expressing rhesus macaque CD16 at a 10:1 effector-to-target ratio in the presence of serial dilutions of plasma [85]. Non-specific lysis was detected as a reduction in background luciferase activity (% RLU) for target cells incubated with NK cells in the presence compared to the absence of plasma. Plasma samples that directed ADCC against uninfected cells were depleted of anti-human antibodies by repeated cycles of incubation with CEM.NKR-CCR5-sLTR-Luc cells, followed by centrifugation and plasma transfer, until ADCC responses to uninfected cells were no longer detectable.

To measure ADCC activity in plasma of vaccinated animals, CEM.NKR-CCR5-sLTR-Luc target cells were infected with SIVmac239 or SHIV_AD8-EO_ (internal negative control) by spinoculation for 3 h at 1200 × g in the presence of 40 μg/ml polybrene (EMD Millipore). Four days post-infection, target cells were incubated with the NK cell line KHYG-1 at a 10:1 effector-to-target ratio in the presence of serial plasma dilutions. Luciferase activity was measured after 8 h using the britelite plus luciferase assay system (PerkinElmer). Triplicate wells were tested at each plasma dilution, and wells containing effector cells incubated with uninfected or infected target cells in the absence of plasma were used to determine background and maximal luciferase activity, respectively. ADCC responses were calculated from the dose-dependent loss of luciferase activity in the presence of plasma relative to background and maximal luciferase control wells.

### Pseudovirus neutralization assays

Replication incompetent SIVmac239 pseudovirus was produced by co-transfecting *env* plasmids with an *env*-deficient backbone plasmid (pSG3Δ*env*) in HEK293T cells in a 1:2 ratio, using the X-tremeGENE 9 transfection reagent (Roche). Pseudovirus was harvested after 72 h by sterile-filtration (0.22 μm) of cell culture supernatants, and neutralization was tested by incubating pseudovirus and serum for 1 h at 37°C before transferring them onto TZM-bl cells as previously described [86]. Neutralization was measured in duplicate wells within each experiment. Neutralization was tested starting at 1:10 serum dilutions followed by nine serial 3-fold dilutions to ensure highest sensitivity and range of detection. Neutralization IC_50_ titers were calculated using the ‘One site—Fit logIC50’ regression in Graphpad Prism v7.0. We could not detect vaccine-induced nAb titers against SIVmac239 pseudovirus in any of the monkeys in Groups 1 and 2 at the time of the first SIV challenge.

### Statistics

The non-parametric Kruskal-Wallis test was used to compare the total magnitude of vaccine-induced SIV-specific T-cell responses among the four vaccinated groups. In instances of significant Kruskal-Wallis tests, pairwise Mann-Whitney U tests were used to identify the difference between any two groups. The Kaplan-Meier method and log-rank test were used to determine if any of the four vaccine regimens employed here affected acquisition of SIV infection. For this analysis, the time-to-productive infection was analyzed using the Kaplan-Meier method and the differences between each of Groups 1–4 and the control Group 5 were evaluated using log-rank tests. The Mann-Whitney U test was also used to determine the efficacy of each vaccine regimen in reducing viral replication. Peak and setpoint viral loads were compared between each of Groups 1–4 and the control Group 5. Setpoint VL were calculated as the geometric mean of VLs measured within wks 8–20 PI. Lastly, the Spearman rank correlation was used to indicate immune correlates of protection.

## Supporting information

S1 Fig Undetectable vaccine-induced CD8+ T-cell responses against Vpr, Vpx, and Pol in the three "difficult-to-infect" Group 4 vaccinees. Three days after the 8^th^ IR SIVmac239 challenge, we ordered blood from the Group 4 vaccinees r08031, r06029, and rhBF24 and set up an ICS assay, as described in the Materials and Methods. The stimuli consisted of pools of SIV peptides spanning proteins that were not delivered by the vaccine (e.g., Vpr, Vpx, and Pol), as well as Vif, which was encoded in the immunization protocol. These pools spanned the entire open reading frames of the Vif, Vpr, and Vpx proteins, and aa 1–354, 344–700, and 690–1060 of Pol. Unstimulated cells served as the negative control while PBMC treated with PMA/Ionomycin served as the positive control. Each row corresponds to one monkey and the graphs show percentages of live CD14–CD16–CD20–CD3+CD8+ lymphocytes producing both IFN-γ and CD69.
(PDF)

S2 Fig Vaccine-induced neutralizing antibodies against SIVmac239 are undetectable in macaques in Groups 1 and 2. Sera from animals in Group 1 (A) and Group 2 (B) collected at the time of the first SIV challenge were screened for neutralizing activity against SIVmac239 using a standard TZM-bl assay (see Materials and methods). It was not possible to generate a best-fit curve for r04074 using nonlinear regression. C) Macaque rhBB35 was infected with SIVmac239 as part of another experiment conducted in the Watkins lab and developed neutralizing antibodies against this virus. Serum from this animal was used as the positive control for this assay.
(PDF)
