# Supplementary material for: Vaccine-induced immune responses against both Gag and Env improve control of simian immunodeficiency virus replication in rectally challenged rhesus macaques
Source: PLoS Pathog. 2017 Jul 21;13(7):e1006529. doi: 10.1371/journal.ppat.1006529 (PMC5540612; doi:10.1371/journal.ppat.1006529)
Supplement: S1 Fig — Three days after the 8th IR SIVmac239 challenge, we ordered blood from the Group 4 vaccinees r08031, r06029, and rhBF24 and set up an ICS assay, as described in the Materials and Methods. The stimuli consisted of pools of SIV peptides spanning proteins that were not delivered by the vaccine (e.g., Vpr, Vpx, and Pol), as well as Vif, which was encoded in the immunization protocol. These pools spanned the entire open reading frames of the Vif, Vpr, and Vpx proteins, and aa 1–354, 344–700, and 690–1060 of Pol. Unstimulated cells served as the negative control while PBMC treated with PMA/Ionomycin served as the positive control. Each row corresponds to one monkey and the graphs show percentages of live CD14–CD16–CD20–CD3+CD8+ lymphocytes producing both IFN-γ and CD69. (PDF) [file ppat.1006529.s001.pdf]

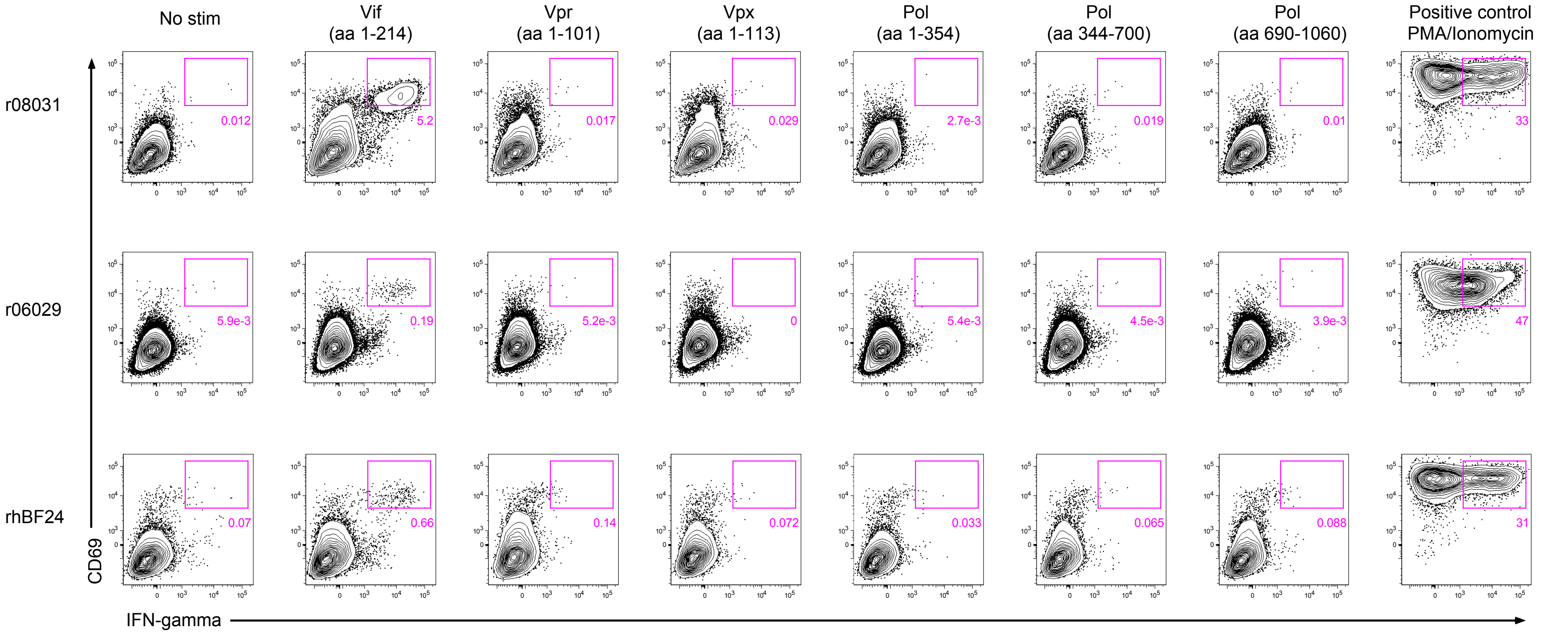

S1 Figure. Undetectable vaccine-induced CD8+ T-cell responses against Vpr, Vpx, and Pol in the three "difficult-to-infect" Group 4 vaccinees. Three days after the 8th IR SIVmac239 challenge, we ordered blood from the Group 4 vaccinees r08031, r06029, and rhBF24 and set up an ICS assay as described in the Materials and Methods. The stimuli consisted of pools of SIV peptides spanning proteins that were not delivered by the vaccine (Vpr, Vpx, and Pol), as well as Vif, which was encoded in the immunization protocol. These pools spanned the entire open reading frames of the Vif, Vpr, and Vpx proteins, and amino acids (aa) 1-354, 344-700, and 690-1060 of Pol. Unstimulated cells served as the negative control while PBMC treated with PMA/Ionomycin served as the positive control. Each row corresponds to one monkey and the graphs show percentages of live CD14-CD16-CD20-CD3+CD8+ lymphocytes producing both IFN-gamma and CD69.
