# Supplementary material for: Vaccine-induced immune responses against both Gag and Env improve control of simian immunodeficiency virus replication in rectally challenged rhesus macaques
Source: PLoS Pathog. 2017 Jul 21;13(7):e1006529. doi: 10.1371/journal.ppat.1006529 (PMC5540612; doi:10.1371/journal.ppat.1006529)
Supplement: S2 Fig — Sera from animals in Group 1 (A) and Group 2 (B) collected at the time of the first SIV challenge were screened for neutralizing activity against SIVmac239 using a standard TZM-bl assay (see Materials and methods). It was not possible to generate a best-fit curve for r04074 using nonlinear regression. C) Macaque rhBB35 was infected with SIVmac239 as part of another experiment conducted in the Watkins lab and developed neutralizing antibodies against this virus. Serum from this animal was used as the positive control for this assay. (PDF) [file ppat.1006529.s002.pdf]

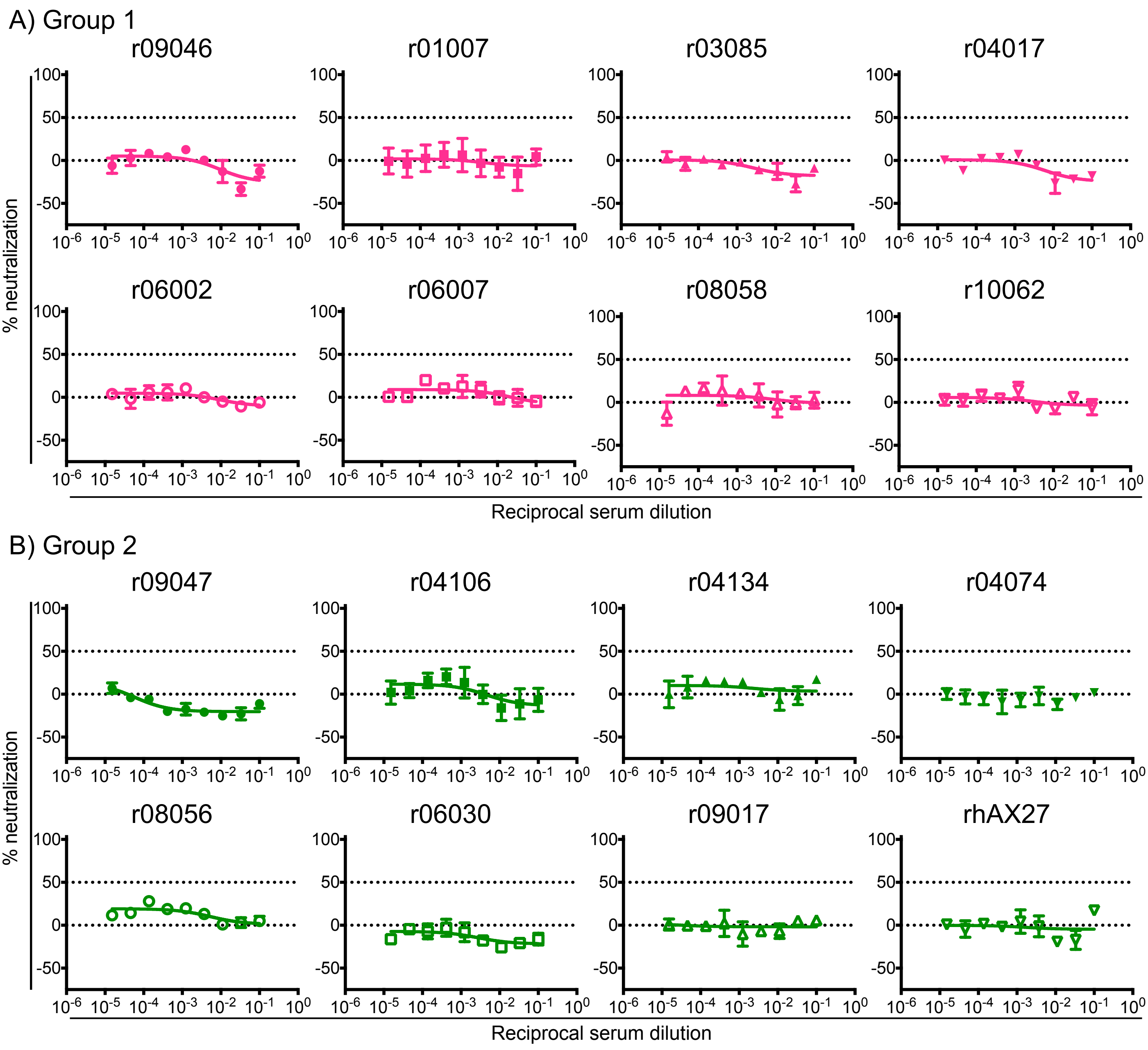

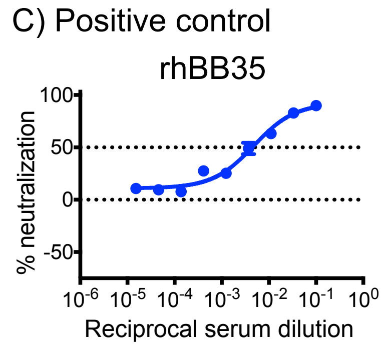

S2 Figure. Vaccine-induced neutralizing antibodies against SIVmac239 are undetectable in macaques in Groups 1 and 2. Sera from animals in Group 1 (A) and Group 2 (B) collected at the time of the first SIV challenge were screened for neutralizing activity against SIVmac239 using the standard TZM-bl assay (see Materials and Methods). It was not possible to generate a best fit curve for r04074 using nonlinear regression. C) Macaque rhBB35 was infected with SIVmac239 as part of another experiment conducted in the Watkins lab and developed neutralizing antibodies against this virus. Serum from this animal was used as the positive control for this assay.
